# Emerging roles of Notch signaling in the tumor microenvironment of digestive system cancers

**DOI:** 10.3389/fmolb.2026.1840769

**Published:** 2026-06-17

**Authors:** Qingmiao Shi, Shuwen Yang, Leiya Fu, Chen Xue

**Affiliations:** 1 Department of Infectious Diseases, The First Affiliated Hospital, College of Clinical Medicine, Henan University of Science and Technology, Luoyang, China; 2 Henan Medical Key Laboratory of Gastrointestinal Microecology and Hepatology, Henan University of Science and Technology, Luoyang, Henan, China; 3 Department of Infectious Diseases, The First Affiliated Hospital of Zhengzhou University, Zhengzhou, Henan, China

**Keywords:** digestive system cancer, immunosuppression, Notch signaling pathway, targeted therapy, tumor microenvironment

## Abstract

The Notch signaling pathway is an evolutionarily conserved cell-cell communication cascade with central roles in the development and progression of digestive system cancers. Beyond its established functions in tumor-cell proliferation and stemness, growing evidence indicates that Notch signaling also remodels the tumor microenvironment (TME). This review synthesizes the multifaceted roles of Notch signaling in the TME of digestive system malignancies. We discuss how aberrant Notch activity regulates the recruitment, polarization, and function of key tumor-infiltrating immune cells, including myeloid-derived suppressor cells (MDSCs), tumor-associated macrophages (TAMs), tumor-associated neutrophils (TANs), regulatory T cells (Tregs), and dendritic cells (DCs), thereby fostering an immunosuppressive milieu. Notch signaling also drives the activation and sustained pro-tumorigenic activity of cancer-associated fibroblasts, promotes tumor angiogenesis, and contributes to extracellular matrix remodeling. Together, these effects establish a TME that supports digestive system cancer progression and therapy resistance. Given its central role in TME regulation, the Notch pathway represents a promising therapeutic target. We summarize current strategies, including Notch inhibitors and their combinations with immunotherapies, angiogenesis inhibitors, and other targeted therapies, that aim to overcome treatment resistance and improve clinical outcomes. A clearer understanding of Notch signaling in the TME will be essential for translating these insights into more effective therapies and improving prognosis in patients with digestive system cancers.

## 1 Introduction

Digestive system cancers impose a substantial global health burden. Esophageal, gastric, colorectal, liver, and pancreatic cancers together accounted for 26.3% of cancer cases and 35.4% of cancer-related deaths worldwide in 2018 (Lu et al., 2021; Zhou et al., 2024). The rising incidence of colorectal cancer (CRC), the third most common cancer worldwide in 2022, together with the persistently high mortality of gastric and esophageal cancers, underscores the need to clarify the molecular mechanisms that drive digestive system cancers and to develop more effective therapeutic strategies (Arnold et al., 2020; Collaborators, 2025; Yu et al., 2024).

Tumor initiation and progression are no longer viewed as processes dictated solely by malignant cells (Goenka et al., 2023). Increasing evidence shows that tumor cells engage in complex crosstalk with the surrounding tumor microenvironment (TME), and defining the mechanisms that govern this communication has become an important research priority (Gou et al., 2025; Hanahan, 2022). In the classic seed-and-soil analogy, tumor cells are the seeds and the TME is the soil. The TME is a complex, highly organized, and dynamic ecosystem that supports digestive system cancer progression (Ma et al., 2025). It contains diverse tumor-infiltrating immune cells, cancer-associated fibroblasts, vascular components, extracellular matrix constituents, cytokines, and chemokines that interact dynamically with tumor cells (Binnewies et al., 2018; Ma et al., 2025). This cellular and molecular ecosystem regulates tumor growth, angiogenesis, invasion, metastasis, and response to therapy (Aliazis et al., 2025). Recent studies further show that the immune landscape within the digestive system TME is highly heterogeneous, with distinct spatial and functional immune niches that can restrain or promote tumor progression (Chen H. et al., 2025; Wu et al., 2022a). This complexity highlights the need to identify the signaling pathways that orchestrate cell-cell communication within the TME (Manohar, 2024; Xia et al., 2022). Among these pathways, Notch signaling has emerged as a key regulatory cascade in both physiological and pathological TME contexts. It is an evolutionarily conserved pathway that controls cell fate determination, proliferation, differentiation, apoptosis, and tissue homeostasis across species (Artavanis-Tsakonas et al., 1999; Siebel and Lendahl, 2017; Xu et al., 2024). Accumulating evidence indicates that aberrant Notch signaling contributes to the initiation and progression of digestive system cancers through context-dependent oncogenic or tumor-suppressive functions (Shi et al., 2024). Beyond its direct effects on tumor cells, Notch signaling is increasingly recognized as a major modulator of the TME and its multiple components, thereby promoting tumor progression and heterogeneity (Sun J. et al., 2025).

Although Notch signaling also shapes the TME in lung, breast, brain, and other malignancies (Chen et al., 2026), this review focuses on digestive system cancers for several related reasons. First, as noted above, these cancers represent a major global health burden, and a clearer understanding of their pathogenesis is essential for therapeutic progress. Second, many digestive system cancers share a common endodermal origin (Rodrigues et al., 2018) and often arise from chronic inflammatory or metaplastic conditions within the gastrointestinal tract (Kunze et al., 2021; Quante et al., 2012). A similar molecular link between chronic inflammation and tumorigenesis has recently been reported in bladder cancer (Wang J. et al., 2026). This inflammatory setting is one in which Notch signaling has fundamental roles in stem-cell maintenance, lineage commitment, and tissue homeostasis (Vooijs et al., 2011). Third, the gastrointestinal tract and associated glands harbor a particularly complex and uniquely conditioned TME. Continuous exposure to luminal antigens, the microbiota, and digestive enzymes can modulate Notch ligand expression and downstream signaling in epithelial and stromal compartments (Gou et al., 2024). These features create organ-specific Notch-dependent crosstalk that cannot be readily generalized from studies of other tumor types.

Against this background, this review examines Notch-driven TME interactions in digestive system cancers, with particular attention to immune regulation, stromal crosstalk, and therapeutic implications. A deeper understanding of these processes may support emerging strategies that target the TME and improve clinical outcomes for patients with digestive system malignancies.

## 2 The Notch signaling pathway in digestive system cancers

The Notch signaling pathway is an evolutionarily conserved intercellular communication system that mediates direct interactions between adjacent cells (Zhou et al., 2022). Because Notch signaling has essential roles in development and tissue homeostasis, aberrant activation or suppression of the pathway, through canonical or non-canonical mechanisms, can contribute to human malignancies, including cancers of the digestive system.

### 2.1 Canonical Notch signaling and its dual role in digestive system cancers

Notch signaling operates through direct cell-cell interactions. Transmembrane Notch receptors (Notch1-4 in mammals) bind Delta/Serrate/Lag-2 (DSL) family ligands, including Delta-like and Jagged proteins, on neighboring cells (Gordon et al., 2008; Sprinzak and Blacklow, 2021). Ligand binding triggers a proteolytic cascade that culminates in gamma-secretase-mediated cleavage and release of the Notch intracellular domain (NICD) (Kopan and Ilagan, 2009). NICD then translocates to the nucleus, where it forms a transcriptional activation complex with recombination signal binding protein for immunoglobulin kappa J region (RBPJ) and co-activators such as MAML, displacing co-repressors (Steinbuck and Winandy, 2018). This complex induces target genes, including members of the Hairy and enhancer of split (HES) and Hairy/enhancer-of-split related with YRPW motif (HEY) families, which encode basic helix-loop-helix transcriptional repressors that regulate differentiation and proliferation in diverse cell lineages (Davis and Turner, 2001).

Dysregulated Notch signaling has been implicated in the pathogenesis of digestive system cancers, where it can act in a context-dependent manner as either an oncogene or a tumor suppressor (Liu et al., 2024; Shi et al., 2024). In hepatocellular carcinoma (HCC), for example, hypoxia-induced DTL-mediated degradation of SLTM relieves transcriptional repression of the Notch1 gene. This upregulates Notch1 expression and promotes tumor-cell proliferation, metastasis, and epithelial-mesenchymal transition (EMT) (Chen et al., 2024). Ubiquitin-conjugating enzyme E2 C (UBE2C), which is highly expressed in tumor tissues and is closely associated with malignant progression of HCC (Zhan et al., 2024), has also been reported to activate Notch signaling by upregulating NICD1 and activating the RBPJ K luciferase reporter. In gastric cancer (GC), Notch signaling contributes to tumorigenesis and progression by promoting cancer stemness (Peng et al., 2025), enhancing cell proliferation and survival, and facilitating immune escape (Jiang et al., 2024). In contrast, evidence for a tumor-suppressive role comes from Kras-driven GC models, in which Notch pathway downregulation causes loss of the Jagged1 (JAG1) ligand and the Mist1 marker at gland bases, promoting hyperplasia and metaplasia (Chung et al., 2019). A similar effect has been reported in pancreatic ductal adenocarcinoma (PDAC) (Hanlon et al., 2010), further supporting a tumor-suppressive role for Notch in Kras-induced tumorigenesis. The Delta-like 4 (DLL4)/Notch1 and JAG1/Notch2 axes can also have opposing effects during HCC development (Xiang et al., 2026). DLL4 knockout in liver cancer can render Notch1 signaling ineffective and inhibit tumor development. Conversely, JAG1 deletion leads to aberrant DLL4 upregulation in hepatocytes and disrupts Notch2 signaling, thereby promoting hepatocarcinogenesis (Nakano et al., 2022).

### 2.2 Non-canonical Notch signaling and its contribution to therapy resistance

Beyond the canonical RBPJ-dependent transcriptional program, emerging evidence has identified several non-canonical Notch signaling modalities (Majumder et al., 2021). These modalities can be grouped into three broad categories. First, ligand-independent activation can occur through intracellular trafficking of Notch receptors, which has an important role in ligand-independent receptor activation (Hounjet and Vooijs, 2021). Under certain conditions, other signaling pathways can also activate Notch receptors through crosstalk mechanisms. Second, RBPJ-independent transcriptional regulation is the most extensively studied form of non-canonical Notch signaling and is particularly relevant to digestive system cancers. For example, atypical Notch signaling ligands can transmit signals to the CSL-NICD-Deltex complex, which promotes progenitor-cell differentiation in the gastrointestinal tract through MAG transcriptional activation (Katoh and Katoh, 2007). Notch signaling can also interact with mTOR to stimulate cell growth and cooperate with Wnt and JAK-STAT pathways to drive the malignant phenotype of gastric cancer (Hibdon et al., 2019). Third, activation through non-DSL ligands provide another route. Delta-like 1 homolog (DLK1), a non-canonical Notch ligand, is proposed to interact with the Notch1 receptor and inhibit canonical Notch signaling (Anitua and Alkhraisat, 2019). Growing evidence links DLK1 expression in cancer to worse prognosis and suggests that it may mark cancer stem cells (Pittaway et al., 2021). Recent research indicates that, in adrenocortical carcinoma and small cell lung cancer, DLK1 upregulates ABCB1 (P-gp) through Notch1-mediated signaling and maintains a dedifferentiated tumor-cell state (Sun N. Y. et al., 2025). On the basis of this mechanism, a DLK1-targeted antibody-drug conjugate has entered a phase I clinical trial (NCT06041516).

Clinical trials of gamma-secretase inhibitors (GSIs) in digestive system malignancies have shown limited single-agent activity (Ayaz and Osborne, 2014). Non-canonical Notch signaling offers one mechanistic explanation for this limitation. Although GSIs block the S3/gamma-secretase cleavage step, Notch signaling may still be activated through the non-canonical mechanisms described above and through crosstalk with other pathways. This provides a theoretical rationale for both GSI resistance and the combined use of Notch inhibitors with inhibitors of other pathways, as discussed in Section 4.4.

**FIGURE 1:**
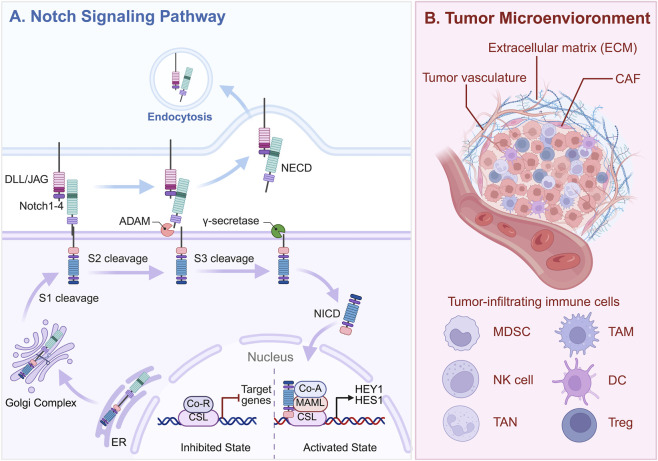
Schematic representation of Notch signaling pathway and the tumor microenvironment. **(A)** In the canonical Notch signaling pathway, ligand-receptor interaction between adjacent cells triggers a series of modifications and cleavages, releasing the NICD, which translocates to the nucleus to regulate the expression of downstream target genes. Non-canonical branches are omitted for clarity and are detailed in the text. **(B)** The tumor microenvironment comprises various cellular and structural components, including tumor vasculature, CAFs, ECM, and diverse tumor-infiltrating immune cells. (Figure created using BioRender.com). DLL Delta-like ligand, JAG Jagged, ADAM a disintegrin and metalloprotease, NECD Notch extracellular domain, NICD Notch intracellular domain, CSL CBF1/suppressor of hairless/Lag1, Co-R corepressor, ER endoplasmic reticulum, Co-A coactivator, MAML mastermind-like transcriptional coactivator, HES1 hairy and enhancer of split 1, HEY1 hairy/enhancer-of-split related with YRPW motif 1, ECM extracellular matrix, CAF cancer-associated fibroblast, MDSC myeloid-derived suppressor cell, NK cell natural killer cell, TAN tumor-associated neutrophil, TAM tumor-associated macrophage, DC dendritic cell, Treg regulatory T cell.

Notch signaling not only responds to intercellular interactions but also integrates microenvironmental signals, enabling cells to sense and respond to their local conditions (LaFoya et al., 2016). When the molecular mechanisms by which Notch contributes to digestive system tumorigenesis and progression are considered, the TME emerges as a major influence at multiple levels (Figure 1). Understanding how microenvironmental cues intersect with canonical and non-canonical Notch pathways is therefore essential for deciphering the context-dependent behavior of Notch in digestive cancers. The following section examines this relationship in detail.

## 3 Roles of Notch signaling in TME remodeling

Notch signaling has a central role in remodeling the TME in ways that favor digestive system cancer progression (Table 1). It influences tumor-infiltrating immune cells, often reprogramming them toward immunosuppressive phenotypes that facilitate immune evasion. The pathway also drives the activation and sustained pro-tumorigenic activity of cancer-associated fibroblasts. In addition, Notch signaling promotes tumor angiogenesis and contributes to extracellular matrix remodeling, collectively establishing a TME that supports cancer progression and therapy resistance.

**TABLE 1:** Key mechanisms by which Notch signaling remodels the TME.

TME component	Cancer type	Notch component	Key mechanism	Cell line	References
MDSC	ESCC	Notch1, Notch2	Overexpression of NEDD9 (in ESCC) → CXCL8↑ → recruitment of G-MDSCs↑ → co-culture of G-MDSCs with ESCC cells → activation of the Notch signaling pathway (in ESCC) →NOTCH1/2, HES1/2 expression↑ → NEDD9↑ → stem cell characteristics of the ESCC↑	KYSE70, KYSE450	Yue et al. (2021)
MDSC	CRC	Notch3	The Notch3 signaling pathway is abnormally activated in CRC tissues → tumor cells secrete CSF1, CXCL12, and CCL2↑ → attract macrophages and MDSC↑ → form an immunosuppressive TME → accelerate the progression of CRC	MC38, HCT116	Huang et al. (2023)
TAM	GC	DLL3	TAMs + GC cells → Notch1/2 signaling pathway in cancer cells ↑ → DLL3↑ → degradation of LG3BP and HSPA8↑ → immune stimulating function of macrophages↓	MKN45, BGC823	Zhao et al. (2022)
TAM	HCC	RBPJ	RBPJ cKO → Notch signal↓ → WNT signaling pathway↑ → β-catenin↑→ kclTAM proliferation↑→ IL-10↑, IL-12↓→ immune-suppressive TME formation → HCC progression	Hepa1-6	Ye et al. (2019)
TAN	CRC	NICD1	In the KPN model → activation of the Notch signaling pathway → CXCL5 and other neutrophil chemoattractants↑ → recruitment of neutrophils to the tumor site↑ → neutrophil TGF-β signaling pathway↑ → formation of an immunosuppressive microenvironment → promotion of CRC metastasis	NA	Ruland (2019)
TAN	CRC	NICD1	In the KRAS G12D-driven serrated CRC mouse model → NICD1 ↑ → expression of chemokines such as CXCL5 and TGF-β2 ↑ → infiltration of neutrophils → formation of immunosuppressive environment → acceleration of CRC progression	NA	Jackstadt et al. (2019)
Treg	GC	Notch1, Notch2	Using DAPT to block the Notch signaling pathway → HES1/HES5↓ → FoxP3↓ → Treg inhibitory function↓ → IL-35 secretion↓ → inhibition of GC progression	NA (clinical samples)	Yang et al. (2019)
Treg	GC	Notch3	The expression of NOTCH3 in GC tissues↑ → PD-1↑ → the proliferation and activation of Tregs↑ → secretion of IL-10 and TGF-β↑ → inhibition of CD8^+^ T cells ↑ → tumor immune escape↑ → progression of GC	NA (clinical samples)	Cui et al. (2020)
DC	CRC	Notch2	Fx^−/−^ mice → deficiency in fucosylation → Notch2 signal↓ → the migration of cDC1 towards CCL19 and CCL21↓ and cross-present antigens to CD8^+^ T cells↓ → inflammation-related CRC↑	OP9	Wang et al. (2021)
CAF	GC	JAG1, Notch3	GC cells secret JAG1 → activate Notch3 receptors in CD146+ CAFs → PI3K/Akt signaling pathway↑ → transcription of CD146↑ → secretion of COL4A1↑ → macrophages to polarize towards M2-like phenotype↑ → activity of CD8^+^ T cells↓ → GC immune escape and tumor progression	AGS, MKN45	Chen J. et al. (2025)
CAF	HCC	Notch1	CAFs → secretion of IL-6↑ → Activation of STAT3 (Tyr705) → Notch1/NICD/HES1↑ → promotion of HCC stem cell characteristics	PLC/PRF/5, MHCC-97H, HLE	Xiong et al. (2018)
Tumor vasculature	ESCC	Notch1	Notch1 ligand binding → NICD1↑ → formation of NICD1-RBPJ complex → USP5 gene transcription↑ → deubiquitination and stabilization of STAT3 → secretion of pro-angiogenic factors (VEGF, ANGPT2, CXCL1) ↑ → migration of endothelial cells and formation of lumen↑ → tumor angiogenesis↑ → acceleration of ESCC growth and metastasis	KYSE30, KYSE510, KYSE450, KYSE150	Li et al. (2025)
Tumor vasculature	GBC	Notch1	GBC cells secrete exosomes containing TRPM2-AS → exosomes are taken up by HUVECs → the inhibition of PABPC1 on NUMB mRNA↑ → NUMB↓ → Notch1 signaling pathway↑→the proliferation, migration and tube formation ability of HUVECs↑ → tumor angiogenesis↑ → the growth and metastasis of GBC↑	GBC-SD, NOZ, SGC-996, EH-GB1	He et al. (2024)
ECM	CRC	HES1	KRAS mutation → abnormal Notch signaling pathway → HES1 expression deficiency → ECM degrading enzymes↓, ECM degrading enzymes such as MMPs↑ → ECM remodeling and degradation↑ → invasion and metastasis ability of CRC cells↑	SW620	Wang et al. (2023)

### 3.1 Modulating tumor-infiltrating immune cells

**FIGURE 2:**
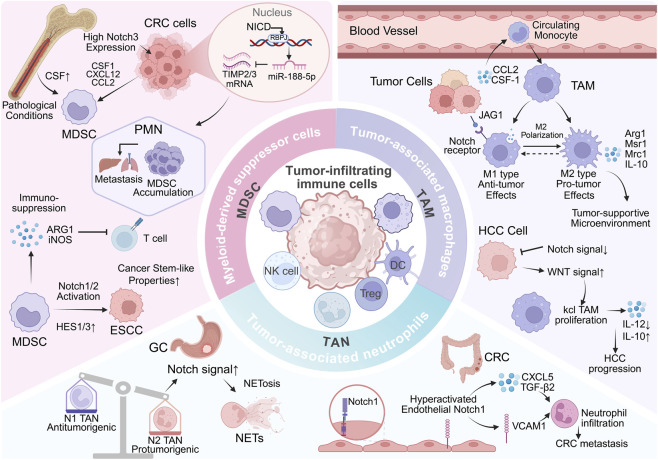
The role of Notch signaling pathway in modulating tumor-infiltrating immune cells. Notch signaling pathway profoundly influences tumor-infiltrating immune cells including MDSCs, TAMs, TANs and so on, often reprogramming them towards immunosuppressive phenotypes that facilitate immune evasion and accelerate digestive system cancers progression. (Figure created using BioRender.com). CRC colorectal cancer, RBPJ recombination signal binding protein for immunoglobulin kappa J region, CSF colony-stimulating factor, CXCL12 C-X-C motif chemokine ligand 12, CCL2 C-C motif chemokine ligand 2, TIMP2/3 tissue inhibitor of metalloproteinases 2/3, PMN pre-metastatic niche, Arg1 arginase 1, Msr1 macrophage scavenger receptor 1, Mrc1 mannose receptor C-type 1, HCC hepatocellular carcinoma, iNOS inducible nitric oxide synthase, NK cell natural killer cell, DC dendritic cell, ESCC esophageal squamous cell carcinoma, GC gastric cancer, NETosis neutrophil extracellular trap formation, NETs neutrophil extracellular traps, VCAM1 vascular cell adhesion molecule 1.

Under physiological conditions, immune cells within the TME can rapidly recognize and eliminate tumor cells (Hanahan and Weinberg, 2011; Xiang et al., 2021). Tumor cells, however, can evade recognition and lysis through diverse mechanisms, promoting immune escape and immune tolerance. Tumor-infiltrating immune cells are key TME components and include myeloid-derived suppressor cells, tumor-associated macrophages, tumor-associated neutrophils, regulatory T cells, dendritic cells, and other populations. These cells interact directly or indirectly with tumor cells through Notch signaling pathways (Figure 2), thereby modulating tumor-cell behavior (Clara et al., 2020; Khalaf et al., 2021).

#### 3.1.1 Myeloid-derived suppressor cells (MDSCs)

MDSCs are a major immunosuppressive myeloid population and are important in TME formation (Clara et al., 2020). They comprise heterogeneous immature myeloid cells with arrested differentiation. The major bone marrow (BM)-derived myeloid populations include granulocytes, mainly neutrophils, and mononuclear cells, including monocytes, terminally differentiated macrophages (MΦ), and dendritic cells (DCs) (Veglia et al., 2018). Under physiological conditions, colony-stimulating factors (CSFs) promote normal immune-cell differentiation, support specific immune functions, and maintain organismal homeostasis (Schulz et al., 2012; Wu et al., 2022b). In pathological contexts such as the TME, these factors are overproduced and disrupt normal myelopoiesis, favoring the expansion of immature MDSCs. These cells mediate immunosuppression and also directly support tumor growth, cancer stemness or dedifferentiation, and metastasis through multiple mechanisms (Marvel and Gabrilovich, 2015; Nakano et al., 2022). The role of Notch signaling in this process is only beginning to be defined.

MDSCs can enhance stemness features in esophageal squamous cell carcinoma (ESCC) cells. Yue et al. used RT-qPCR to show that co-culture of ESCC cells with MDSCs significantly increased the transcriptional levels of key Notch pathway components, including Notch1/2 and HES1/3, indicating that MDSCs enhance cancer stem-like properties in ESCC through the Notch pathway (Yue et al., 2021). MDSCs also expand substantially and exert immunosuppressive effects in CRC (Shi et al., 2025; Sieminska and Baran, 2020). Huang and colleagues found that Notch3 signaling is highly expressed in CRC. Gene set enrichment analysis (GSEA) showed a significant positive correlation between Notch3 expression and MDSC-recruiting cytokines (CSF1, CXCL12, and CCL2), suggesting that Notch3 promotes CRC progression by increasing MDSC infiltration (Huang et al., 2023). In addition, Notch signaling transcriptionally activates miR-188-5p through RBPJ binding to its promoter. This microRNA downregulates tissue inhibitors of metalloproteinases 2 and 3 (TIMP2 and TIMP3), which are key regulators of ECM integrity and immune function. The result is formation of a pre-metastatic niche (PMN), marked by extensive MDSC recruitment to the liver and lungs, that ultimately drives CRC metastasis (Wang Y. et al., 2026). The interplay between Notch activation and MDSC recruitment or function therefore highlights a potential therapeutic vulnerability. Targeting Notch signaling could disrupt MDSC-driven immunosuppression and metastasis and may support combined immunotherapeutic strategies.

#### 3.1.2 Tumor-associated macrophages (TAMs)

TAMs, which derive from circulating monocytes recruited to tumor sites by chemotactic signals such as CCL2 and CSF-1, are pivotal TME components. They are generally divided into two functionally contrasting subtypes: M1 macrophages, which exert antitumor effects, and M2 macrophages, which promote tumor occurrence and metastasis (Bied et al., 2023; Xu et al., 2025). Both subtypes are highly plastic and can interconvert in response to TME changes or therapeutic interventions (Pan et al., 2020). Acquisition of an M2-like state is important for establishing a microenvironment that supports cancer-cell survival and progression (Chen et al., 2019). Notch signaling has been shown to govern macrophage maturation into the TAM phenotype, also referred to as M2 macrophages (Liu et al., 2017).

For example, in the pancreatic ductal adenocarcinoma microenvironment, tumor epithelial cells, endothelial cells, and fibroblasts highly express Notch ligands such as JAG1, whereas TAMs highly express Notch receptors (Yan et al., 2024). Notch pathway activation in TAMs promotes M2-like polarization, characterized by high expression of immunosuppressive mediators such as Arg1, Msr1, Mrc1, and IL-10, thereby fostering a tumor-supportive microenvironment. Other studies indicate that Notch signaling can also promote macrophage polarization toward the M1 phenotype (Wang et al., 2020; Zhao et al., 2022). In GC, co-culture of TAMs with MKN45 or BGC823 GC cells activated Notch1/2 signaling in the cancer cells and increased DLL3 expression (Zhao et al., 2022). DLL3 overexpression resulted in degradation of LG3BP and HSPA8, both of which have immunostimulatory functions in macrophages, potentially suppressing macrophage immune activity.

Notch signaling can also interact with other pathways to regulate TAM proliferation and differentiation. In HCC, Notch blockade enhances WNT signaling. Through a beta-catenin-dependent mechanism, this promotes proliferation of kclTAMs (Kupffer cell-like TAMs), upregulates IL-10, and downregulates IL-12, thereby creating an immunosuppressive TME (Ye et al., 2019). In summary, Notch pathway activation in cancer cells can affect the antitumor immune function of macrophages. Conversely, cancer cells can activate Notch signaling in macrophages and drive acquisition of an M2 phenotype. Future studies should further define the interplay between Notch signaling and other pathways in TAMs and develop corresponding targeted therapies.

#### 3.1.3 Tumor-associated neutrophils (TANs)

Neutrophils, the most abundant circulating white blood cells, occupy a crucial position in the TME and provide an important link between inflammation and cancer progression (Wu et al., 2019). Within the TME, TANs show functional plasticity and can adopt two predominant polarization states: the antitumorigenic N1 phenotype and the pro-tumorigenic N2 phenotype. The balance between these phenotypes is dynamically regulated by contextual cues in the TME, allowing reversible phenotypic switching and functional adaptation (Que et al., 2022; Zhou Y. et al., 2025). N2-polarized TANs contribute to tumor progression and metastasis, partly through neutrophil extracellular trap (NET) formation (Adrover et al., 2023). In highly aggressive GC, for example, immature but highly active neutrophils show elevated expression of NETosis-associated genes and increased Notch pathway activation, suggesting that Notch signaling may regulate neutrophil function and contribute to a pro-metastatic TME (Ma et al., 2024; Qi et al., 2025).

Across diverse human tumors and PMNs, endothelial NICD1 is frequently hyperactivated. This induces endothelial senescence and upregulates chemokines and the adhesion molecule VCAM1, facilitating neutrophil infiltration, tumor-cell adhesion, and metastasis (Wieland et al., 2017). In CRC, pathological activation of Notch1 signaling in tumor epithelial cells upregulates chemokines, including CXCL5 and TGF-β2, thereby recruiting neutrophils and initiating a TGF-β-mediated, neutrophil-dependent cascade that drives tumor metastasis (Jackstadt et al., 2019; Wieland et al., 2017). In the KPN model, a metastatic CRC model induced by Kras G12D activation, Trp53 inactivation, and NICD1 expression, neutrophil-related genes are significantly enriched (Ruland, 2019). Most current studies have focused on experimentally forced activation of Notch1 signaling to drive high-penetrance metastasis *in vivo*. However, Notch1 mutations are rare in human solid tumors. Further research is therefore needed to identify the main mechanisms underlying excessive Notch activation in human tumor cells and the distinct contributions of individual Notch proteins to tumor onset and metastasis.

#### 3.1.4 Regulatory T cells (tregs)

Tregs are a subset of CD4^+^ T cells with immunoregulatory functions and are typically identified as CD4^+^CD25+FoxP3+ cells (Imianowski et al., 2025). On the basis of origin, Tregs can be divided into thymus-derived natural Treg cells (tTregs) and peripherally induced Treg cells (pTregs), which arise from conventional CD4^+^ T cells after TCR stimulation in the presence of TGF-beta and IL-2 (Tan et al., 2025). Notch and TGF-beta signaling have been reported to play major roles in differentiation of naive T cells into pTregs (Trehanpati and Vyas, 2017). Numerous studies have shown that tumor-infiltrating Tregs are increased in many cancers (Fu et al., 2007; Shah et al., 2011). The development and differentiation of these cells are influenced by multiple signaling pathways, especially the Notch pathway, and are closely linked to digestive system tumors (Gragnani et al., 2020; Wei et al., 2023).

In patients with HBV-related hepatocellular carcinoma (HBV-HCC), Notch1 expression is significantly upregulated in CD4^+^CD25^+^CD127Low Tregs (Sharma et al., 2015). Flow cytometry and quantitative RT-PCR confirmed activation of Notch signaling in these Tregs, which express higher levels of Forkhead box P3 (Foxp3) and show stronger immunosuppressive capacity. In GC, Yang and co-workers found that Notch1 and Notch2 expression is significantly elevated in GC tissues, and that the proportion of CD4^+^CD25^+^CD127dim/^-^ Tregs in the peripheral blood of patients with GC is markedly increased. Blocking Notch signaling significantly reduced Treg suppressive function and decreased IL-35 production (Yang et al., 2019). Cui et al. further reported that Treg infiltration is significantly increased in the TME of patients with GC and high Notch3 expression (Cui et al., 2020). Mechanistically, Notch signaling may negatively regulate the antitumor activity of CD8^+^ T cells by upregulating immune checkpoint molecules such as programmed cell death protein 1 (PD-1), and this mechanism may also extend to Tregs. Among ligands, overexpression of JAG1 in tumor cells has been shown to induce Treg proliferation and activation (Izadi et al., 2018). Collectively, these studies indicate that Notch pathway activation enhances Treg functional activation, reinforces immunosuppression, and may represent a targetable immunotherapeutic axis.

#### 3.1.5 Dendritic cells (DCs)

As the most potent antigen-presenting cells, DCs have a crucial role in initiating T-cell immune responses against tumors. DC migration from peripheral tissues to secondary lymphoid organs is essential for effective antitumor immunity (Zanna et al., 2021). However, the TME can impair DC migration through several mechanisms and thereby enable tumor evasion of immune surveillance (Youlin et al., 2018). Recent studies provide evidence that Notch signaling regulates DC differentiation and function. Notch signaling is required for cDC1 generation in a time-dependent manner (Balan et al., 2018). In an *in vitro* culture system, DLL1-dependent Notch signaling promotes cDC1 differentiation. Recent work also showed that the carrier protein Hla H35 A targets the ADAM10 receptor on DCs to drive Notch2-dependent differentiation of cDC2s, which express endothelial cell-selective adhesion molecule (ESAM) and secrete IL-23, thereby exerting antitumor effects (Wang et al., 2025). Notch signaling also regulates T-cell polarization by DCs (Cheng et al., 2010). For example, JAG1 directs T-cell differentiation by activating GATA-3 and inhibiting Spi-B. Specific deletion of the Notch ligands DLL1 or JAG2 in DCs showed that DLL1 loss significantly accelerated lung and pancreatic cancer growth (Tchekneva et al., 2019).

In *Fx* ^*−/−*^ mice, deficient fucosylation causes loss of Notch2 signaling, resulting in impaired cDC1 migration toward CCL19 and CCL21 and reduced cross-presentation of antigens to CD8^+^ T cells (Wang et al., 2021). These functional defects promote inflammation-associated CRC. Recent findings by Balan and colleagues further indicate that DLL1-induced cDC1s (DLL1_cDC1s) show higher expression of genes associated with migration, effector-cell interaction, and chemokine production, together with a more activated state (Balan et al., 2025). In a humanized mouse model, injection of tumor-antigen-loaded DLL1_cDC1s combined with PD-1 inhibitors significantly reduced melanoma growth and achieved complete tumor regression in one animal. These findings may inform future approaches for digestive system tumors. Given interpatient heterogeneity in digestive cancers, high-throughput sequencing could be used to identify tumor-specific neoantigens for loading onto DLL1_cDC1s to generate personalized tumor vaccines and potentially minimize off-target effects.

### 3.2 Regulating cancer-associated fibroblast (CAF) activity

**FIGURE 3:**
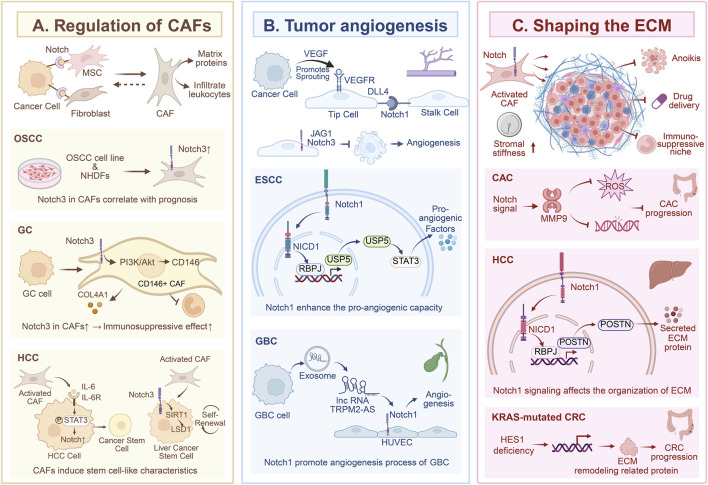
The diverse function of Notch signaling in the tumor microenvironment. **(A)** Notch signaling regulates the differentiation and function of CAFs, influencing immunosuppression and stem cell-like characteristics. **(B)** Notch signaling, particularly through Notch1 and Notch3, promotes tumor angiogenesis by regulating tip/stalk cell dynamics and pro-angiogenic factor expression. **(C)** Notch signaling in CAFs and cancer cells contributes to ECM remodeling, affecting drug delivery, immune niche formation and cancer progression. (Figure created using BioRender.com). MSC mesenchymal stem cell, OSCC oral squamous cell carcinoma, NHDF normal human dermal fibroblast, GC gastric cancer, COL4A1 collagen type IV alpha 1 chain, HCC hepatocellular carcinoma, IL-6R interleukin-6 receptor, VEGF vascular endothelial growth factor, VEGFR vascular endothelial growth factor receptor, DLL4 Delta-like ligand 4, JAG1 Jagged 1, ESCC esophageal squamous cell carcinoma, GBC gallbladder cancer, LncRNA long non-coding RNA, HUVEC Human Umbilical Vein Endothelial Cells, USP5 ubiquitin specific peptidase 5, CAC colitis-associated cancer, MMP9 matrix metallopeptidase 9, ROS reactive oxygen species, CRC colorectal cancer, POSTN periostin.

Tumors can reprogram several cell types into CAFs. Resident fibroblasts and stellate cells in the pancreas and liver are common sources, but other cells, including BM-derived mesenchymal stem cells and cancer cells that have undergone EMT, may also contribute (Chen and Song, 2019; Hinshaw and Shevde, 2019; Peng et al., 2024). These newly formed CAFs have diverse roles within the TME, including extracellular matrix deposition and remodeling, bidirectional signaling with malignant cells, and complex interactions with infiltrating immune cells (Sahai et al., 2020). CAFs are highly heterogeneous and include multiple subpopulations with distinct roles in tumor progression, invasion, and metastasis (Mitriashkin et al., 2025). Signaling cascades act as communication bridges between cancer cells and CAFs (Fang et al., 2023). Fibroblasts can become CAFs through direct physical contact with tumor cells and can be activated through Notch signaling (Park et al., 2020). By altering their transcriptional and secretory profiles, activated CAFs profoundly influence the malignant behavior of digestive system cancer cells. This regulatory effect depends in part on modulation of the Notch signaling pathway (Figure 3) (Zhong et al., 2024).

In oral squamous cell carcinoma (OSCC), Notch3 expression is induced in CAFs and is associated with poor patient prognosis. *In vitro* co-culture experiments confirmed that OSCC cell lines stimulate Notch3 expression in normal human dermal fibroblasts (NHDFs), suggesting that Notch3 expression may serve as a prognostic indicator in OSCC (Kayamori et al., 2016). In GC tissues, tumor cells activate Notch3 in CD146+ CAFs, a distinct CAF subgroup. This activation promotes CD146 transcription through the PI3K/Akt pathway and increases COL4A1 secretion, thereby enhancing the immunosuppressive capacity of these CAFs within the TME (Chen J. et al., 2025). In HCC, CAFs are one of the major TME cell types. Studies show that CAFs can induce stem cell-like characteristics in non-stem cancer cells, allowing them to re-enter a stem-like state (Lau et al., 2016; Liao et al., 2010). Activated CAFs secrete high levels of IL-6, which activates STAT3, phosphorylated at Tyr705, in cancer cells and upregulates Notch signaling, thereby promoting stem-like properties in HCC cells (Xiong et al., 2018). Another study reported that CAFs activate Notch3 signaling, upregulate SIRT1, and subsequently activate lysine-specific demethylase 1 (LSD1). This promotes liver cancer stem-cell self-renewal and tumor formation, accelerating HCC progression (Liu et al., 2018). Notch activation in CAFs also appears to selectively influence autophagy and DNA damage and repair processes (Fang et al., 2023). Together, these findings suggest that targeting Notch signaling may provide a strategy to regulate CAF function and inhibit digestive system cancer progression.

### 3.3 Influencing tumor angiogenesis

Over the past two decades, studies have shown that Notch has a critical role in angiogenesis through highly dynamic and cell-type-specific regulatory mechanisms. As a key pathway controlling vascular endothelial cell fate, Notch precisely regulates vessel initiation and branching through two ligand-receptor axes: DLL4-Notch1 and JAG1-Notch2/3 (Fernández-Chacón et al., 2021). In tumor angiogenesis, DLL4 is highly expressed in tip cells. By activating Notch1 signaling in adjacent stalk cells, DLL4 restricts their proliferative capacity and maintains vascular architecture (Akil et al., 2021; Naito et al., 2020). Blocking DLL4 or Notch1 signaling induces nonproductive angiogenesis and tumor-center necrosis, highlighting the importance of Notch signaling in tumor angiogenesis (Noguera-Troise et al., 2006). For example, selenoprotein1 exerts antiangiogenic effects in CRC by directly interacting with DLL4, suppressing DLL4/Notch1 signaling, and inhibiting tumor blood-vessel formation (Zhang et al., 2022). Notch3 function is also regulated by JAG1 expression levels in the TME. High JAG1 expression inhibits the pro-apoptotic effect of Notch3 and thereby promotes angiogenesis (Lin et al., 2017). The Notch and VEGF pathways also interact in complex ways. VEGF promotes tip-cell formation and migration, whereas Notch signaling coordinates stalk-cell behavior to counterbalance this action and precisely regulate angiogenesis (Pan et al., 2024).

In ESCC, Li et al. used *in vitro* and *in vivo* experiments to show that Notch1 activation significantly enhanced the pro-angiogenic capacity of ESCC cell-conditioned medium (Li et al., 2025). Mechanistically, NICD1 binds RBPJ to upregulate ubiquitin-specific peptidase 5 (USP5), which deubiquitinates and stabilizes STAT3 protein and thereby promotes secretion of pro-angiogenic factors. Another study showed that gallbladder cancer (GBC) cells secrete exosomes containing the long non-coding RNA (lncRNA) TRPM2-AS, which promotes tumor angiogenesis by activating Notch1 signaling in HUVECs. This suggests that combining TRPM2-AS inhibitors with Notch1 inhibitors may represent a future therapeutic strategy for GBC (He et al., 2024). Collectively, this multilevel regulatory network positions Notch signaling as a promising therapeutic target for inhibiting tumor angiogenesis. Clinical translation, however, will require careful balancing of antiangiogenic efficacy against the immunomodulatory consequences of TME remodeling.

### 3.4 Shaping the extracellular matrix (ECM)

In digestive system cancers, Notch signaling has a pivotal role in ECM remodeling and thereby shapes a tumor-promoting microenvironment. Notch activation in CAFs upregulates secretion of ECM components, including collagens and fibronectin, leading to increased stromal stiffness (Song and Zhang, 2018). Beyond regulating CAF function, key Notch pathway components can also directly influence the ECM. For example, in KRAS-mutated CRC, HES1 deficiency, involving a downstream Notch target, activates genes and pathways involved in ECM remodeling and creates favorable conditions for tumor-cell invasion and metastasis (Wang et al., 2023). Kongkavitoon et al. showed that NICD1 directly binds the regulatory region of POSTN, which encodes a secreted ECM protein, indicating that Notch signaling affects ECM organization through transcriptional regulation in HCC (Kongkavitoon et al., 2018). In addition, Notch-regulated MMP9, a zinc-dependent endopeptidase, inhibits colitis-associated cancer (CAC) development by limiting reactive oxygen species (ROS) accumulation and DNA damage, thereby preserving genomic stability (Walter et al., 2020).

This remodeled, rigid ECM does more than provide passive structural support. It activates signaling pathways in cancer cells, enhancing proliferation, invasive capacity, and resistance to anoikis, a form of apoptosis triggered by detachment from the ECM (Wu et al., 2024). By conferring anoikis resistance, the Notch-shaped matrix enables disseminated tumor cells to survive in circulation, a critical step in metastatic colonization (Xiang et al., 2025a). The dense ECM also acts as a physical barrier that impairs drug delivery and fosters an immunosuppressive niche (Callaway et al., 2025; Zhang et al., 2025). Thus, the Notch-ECM axis functions as a central driver of tumor progression, metastasis, and therapy resistance within the gastrointestinal TME.

## 4 Therapeutic strategies targeting TME components through Notch signaling

**FIGURE 4:**
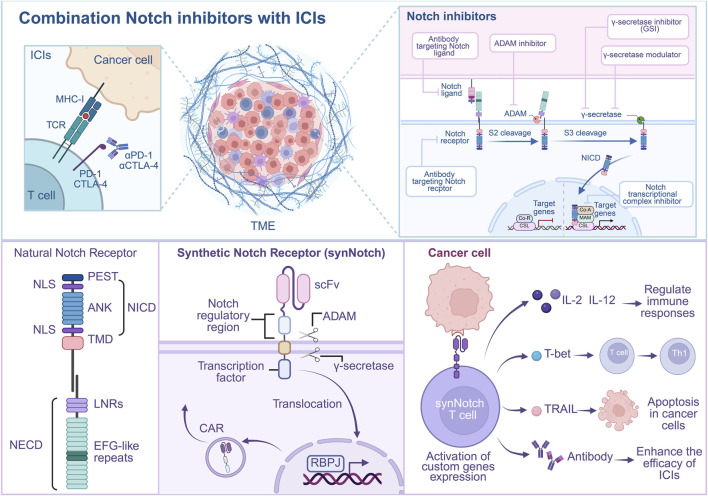
Combination strategies targeting Notch signaling with immunotherapies. The upper panel illustrates the combination of Notch inhibitors with ICIs to enhance anti-tumor immunity. The lower panel details the synNotch receptor based on the structural modification of the natural Notch receptor and the immunological effect exerted by synNotch T cells upon interacting with tumor cells. (Figure created using BioRender.com). ICIs immune checkpoint inhibitors, PD-1 programmed cell death protein 1, CTLA-4 cytotoxic T-lymphocyte-associated protein 4, αPD-1 anti-programmed cell death protein 1 antibody, αCTLA-4 anti-cytotoxic T-lymphocyte-associated protein 4 antibody, MHC-I major histocompatibility complex class I, TCR T cell receptor, SynNotch synthetic Notch receptor, PEST proline/glutamic acid/serine/threonine, ANK ankyrin repeat, TMD transmembrane domain, LNRs Lin12-Notch repeats, EGF epidermal growth factor, NECD Notch extracellular domain, NLS nuclear localization sequences, scFv single-chain fragment variable, CAR chimeric antigen receptor, T-bet T-box transcription factor 21, TRAIL TNF-related apoptosis-inducing ligand.

Notch signaling mediates critical interactions within the TME, making it a promising therapeutic target. Current strategies aim to disrupt communication between cancer cells and TME components, including immune cells, vascular endothelial cells, and the ECM. By modulating the Notch pathway, investigators seek to overcome immunosuppression, normalize tumor vasculature, and inhibit stromal support (Figure 4).

### 4.1 Application of Notch inhibitors

Notch pathway inhibitors, including GSIs, gamma-secretase modulators, ADAM inhibitors, transcriptional complex inhibitors, and receptor- or ligand-specific monoclonal antibodies, provide a strategy for disrupting a signaling cascade that is frequently dysregulated in digestive system tumors (Chen et al., 2026; Zhou et al., 2022). These agents are designed to block Notch-driven oncogenic processes, including sustained proliferative signaling, stemness maintenance (Fender et al., 2015), and therapy resistance (Feng et al., 2024; Yang et al., 2018). Several Notch-targeted agents have entered clinical trials for digestive system cancers (Table 2).

**TABLE 2:** Summary of clinical trials investigating Notch pathway inhibitors in digestive system cancers.

Agent	Target	Cancer type	Phase	Key findings	NCT identifier	Ref
RO4929097	γ-secretase	Metastatic PC	II (completed)	In 18 pretreated metastatic pancreatic cancer patients, RO4929097 was well-tolerated but showed limited activity: 6-month survival rate 27.8%, median OS 4.1 months, median PFS 1.5 months, and 3 of 12 evaluable patients (25%) had stable disease	NCT01232829	De Jesus-Acosta et al. (2014)
RO4929097	γ-secretase	Metastatic CRC	II (completed)	In 33 evaluable patients, RO4929097 showed no objective responses: 6 (18%) had stable disease as best response, with median PFS of 1.8 months and median OS of 6.0 months	NCT01116687	Strosberg et al. (2012)
RO4929097 + Cetuximab	γ-secretase + EGFR	Metastatic CRC	I/II (terminated)	Trial terminated; no efficacy data available	NCT01198535	Strosberg et al. (2012)
RO4929097 + FOLFOX + Bevacizumab	γ-secretase + chemotherapy + VEGF	Metastatic CRC	I (withdrawn)	Trial withdrawn prior to enrollment; no efficacy data available	NCT01270438	Strosberg et al. (2012)
RO4929097 + Capecitabine	γ-secretase + antimetabolite	Refractory solid tumors, including colon cancer	I (completed)	In 30 evaluable patients, the combination was well-tolerated: two confirmed partial responses were observed in patients with fluoropyrimidine-refractory colon cancer	NCT01158274	LoConte et al. (2015)
LY3039478	γ-secretase	Gastrointestinal stromal tumors	I (completed)	In 13 pretreated GIST patients, LY3039478 showed modest activity: 4 (31%) had stable disease (all lasting >3 months), with median PFS of 1.9 months and median OS of 16.5 months	NCT01695005	Mir et al. (2018)
LY3039478	γ-secretase	Advanced solid tumors including CRC and PC	I (completed)	In 16 heavily pretreated CRC patients and 7 PC patients, no objective responses were reported. Overall, single-agent activity was limited in unselected solid tumors, with only one partial response (breast cancer) and several stable disease cases across all tumor types	NCT01695005	Azaro et al. (2021a), Massard et al. (2018)
LY3039478 + Taladegib	γ-secretase + SMO	Advanced solid tumors including PC	Ib (completed)	In the combination with taladegib, no objective responses were observed; the regimen was poorly tolerated due to toxicities	NCT02784795	Azaro et al. (2021b)
LY3039478 + LY3023414	γ-secretase + PI3K/Mtor	Advanced solid tumors including colon cancer	Ib (completed)	In 32 patients (including colon cancer), no partial or complete responses; stable disease rate was 18.8% (6 patients), and the MTD of crenigacestat was 25 mg TIW due to dose-limiting toxicities	NCT02784795	Azaro et al. (2021b)
LY900009	γ-secretase	Advanced solid tumors, including CRC and PC	I (completed)	In 35 patients, no objective responses were observed: 5 (14%) had stable disease, including one patient with rectal carcinoma who remained stable for 55 days. No specific efficacy signals were noted in colorectal or pancreatic subsets	NCT01158404	Pant et al. (2016)
MK-0752	γ-secretase	Advanced solid tumors including digestive system cancers	I (completed)	In 16 pretreated CRC patients, MK-0752 showed no objective responses; essentially no clinical activity was observed in extracranial solid tumors	NCT00106145	Krop et al. (2012)
MK-0752	γ-secretase	PDAC	I (completed)	In 44 patients with stage III/IV PDAC, the combination of MK-0752 and gemcitabine was tolerated at full single-agent RP2Ds: among 19 evaluable patients, 13 had stable disease and 1 achieved a confirmed partial response, but overall activity was similar to gemcitabine alone	NCT01098344	Cook et al. (2018)
BMS-986115	γ-secretase	Advanced solid tumors including digestive system cancers	I (completed)	In 36 pretreated advanced solid tumor patients, including 14 with digestive cancers. Among 11 patients with stable disease, 5 had SD > 6 months; no specific efficacy for digestive tumors was separately reported	NCT01986218	Aung et al. (2018)
Demcizumab (OMP-21M18)	DLL4	Advanced solid tumors including digestive system cancers	I (completed)	In previously treated patients with advanced solid tumors including CRC and PC, demcizumab at 10 mg/kg every other week showed disease stabilization in 64% (16/25) of evaluable patients, with one unconfirmed partial response in PC.	NCT00744562	Smith et al. (2014)
CTX-009	DLL4 + VEGF-A	Advanced solid tumors including CRC and GC	I (completed)	In 45 heavily pretreated patients (42% gastric, 40% colorectal), ES104 was well tolerated with no DLTs	NCT04492033	NA
CTX-009	DLL4 + VEGF-A	Metastatic CRC	II (ongoing)	A Simon two-stage study evaluating CTX-009 monotherapy (10 mg/kg Q2W) in patients with ≥2 prior systemic therapies; primary endpoint is ORR; results pending	NCT05513742	NA
CTX-009	DLL4 + VEGF-A	Biliary tract cancer	II (completed)	In 24 biliary tract cancer patients, CTX-009 plus paclitaxel achieved ORR 37.5%, DCR 91.5%, median PFS 9.4 months, and median OS 12.5 months	NCT04492033	NA
CTX-009 + Paclitaxel	DLL4 + VEGF-A	Biliary tract cancer	II/III (ongoing)	In a prior phase II study of 24 pretreated advanced biliary tract cancer patients (phase II cohort of NCT04492033), CTX-009 plus paclitaxel achieved an overall response rate of 37.5% (63.6% in second-line patients), median PFS 9.4 months, and 1-year OS rate 53%	NCT05506943	Azad et al. (2024)
Brontictuzumab (OMP-52M51)	Notch1	Metastatic CRC	Ib (terminated)	Accurate efficacy and safety data are not available	NCT03031691	NA
Tarextumab (OMP-59R5)	Notch2/3	Metastatic PDAC	II (completed)	In untreated metastatic PDAC patients, adding tarextumab to nab-paclitaxel and gemcitabine did not improve OS (median 6.4 vs. 7.9 months; HR = 1.34) and significantly worsened PFS (3.7 vs. 5.5 months; HR = 1.43, p = 0.04)	NCT01647828	Hu et al. (2019)
CB-103	CSL-NICD	Advanced solid tumors including digestive system cancers	I (terminated)	In the dose-escalation cohorts, 8 patients with metastatic CRC and 1 patient with CCA were enrolled, but no objective responses were reported; overall among all solid tumors, 49% had stable disease with no complete or partial responses	NCT03422679	Hanna et al. (2023)
CBA-1205	DLK1	Advanced solid tumors including digestive system cancers	I (ongoing)	In 22 pretreated Japanese patients with advanced solid tumors, CBA-1205 was well tolerated with no dose-limiting toxicities up to 30 mg/kg; 6 patients (27%) had stable disease lasting >6 months (PFS range: 29–144 weeks), including one pancreatic cancer patient with SD for 47 weeks	NCT06636435	Katsuya et al. (2025), Nakamura et al. (2024)

The potential application of Notch inhibitors extends beyond cancer cells to regulation of the TME. As noted above, Notch signaling is active in multiple stromal components within the TME. Inhibiting the pathway could therefore remodel the TME into a state that is less permissive for tumor growth. For example, Notch inhibitors can modulate the TME by reshaping the secretory profiles of TAMs and CAFs, reducing cytokine and growth-factor secretion that sustains pancreatic cancer stem-cell viability and ultimately impeding PDAC progression (Zhou J. et al., 2025). Recently, Yao et al. identified RIN1, a small molecule that selectively blocks the functional interaction between RBPJ and NICD. RIN1 significantly promotes IL-17 and IFN-gamma secretion by CD4^+^ T cells, enhances T-cell-mediated antitumor immunity, and inhibits EMT in HCC cells (Yao et al., 2025). In CRC, Notch inhibitors have also been reported to reduce immunosuppressive-cell abundance, thereby improving the immune status of the TME and facilitating immune-cell infiltration and activation (Zhou et al., 2026).

Clinical translation of this strategy faces important limitations. The most prominent is on-target gastrointestinal toxicity, which reflects the critical physiological role of Notch in gut epithelial homeostasis (Collins et al., 2021; Hu et al., 2019). In addition, the pleiotropic effects of Notch signaling mean that systemic inhibition can sometimes produce contradictory TME outcomes, potentially disrupting beneficial immune responses (Otani et al., 2022; Sun J. et al., 2025). These challenges indicate that Notch inhibitor monotherapy has a constrained efficacy-toxicity profile. To address these limitations and leverage Notch inhibition for broader tumor control, rational combination strategies have become a major focus. Integrating Notch inhibitors with other modalities may enhance direct antitumor cytotoxicity while normalizing the supportive TME, offering a potentially more effective therapeutic paradigm (Sharma et al., 2026). This topic is discussed below.

### 4.2 Combination with immunotherapy

Immune checkpoint inhibitors (ICIs) are monoclonal antibodies that block inhibitory immune pathways, or checkpoints. Rather than directly killing tumor cells, these agents promote antitumor immunity by enhancing T-cell activation (Hayase and Jenq, 2021). Despite their substantial clinical efficacy, ICI activity in cancer treatment is often limited by diverse resistance mechanisms (Renga et al., 2022). Recent preclinical work has therefore explored combination strategies that pair ICIs with selective Notch inhibitors (Meng et al., 2022; Montagner et al., 2024). Luo et al. analyzed mRNA expression and genomic variation in Notch signaling genes in esophageal, gastric, colon, and rectal adenocarcinomas (GIACs) from the TCGA database and found that patients with high mRNA-score GIACs had poorer responses to ICI treatment (Luo et al., 2025). In OSCC, patients with Notch pathway gene mutations, including NOTCH1 and FBXW7 mutations and NOTCH2NLR mutation, showed high programmed death-ligand 1 (PD-L1) expression, increased CD8^+^ T-cell infiltration, and better clinical responses to ICI treatment (Ogi et al., 2024). Similarly, among patients with CRC treated with ICIs, those with NOTCH-MT status had significantly longer overall survival than those with NOTCH-WT (wild-type) status (Lin et al., 2023). In HCC, deficiency of Schlafen Family Member 11 (SLFN11) activates the Notch pathway and promotes macrophage M2 polarization, thereby reducing ICI efficacy. Combining Notch inhibitors may therefore enhance ICI efficacy in HCC patients with low SLFN11 expression (Zhou et al., 2023). Dai et al. also tested combined DAPT and ICI treatment in a mouse model of pancreatic cancer (PC), where the combination significantly inhibited tumor growth compared with monotherapy. Future studies should evaluate the clinical efficacy of Notch inhibitor-ICI combinations and define differences among Notch inhibitors, as well as optimal dose and treatment duration.

Chimeric antigen receptor T-cell (CAR-T) therapy is another important immunotherapy. It has achieved remarkable success in hematological malignancies, but its efficacy remains limited in solid tumors (Vogt et al., 2025). One major obstacle is the lack of tumor-specific cell-surface antigens, which can lead to on-target, off-tumor toxicity. SynNotch (synthetic Notch) is an engineered receptor in which the EGF-like repeat sequence and intracellular transcriptional regulatory region of the natural Notch receptor are replaced. It is a highly programmable synthetic chimeric receptor that can trigger precise, antigen-dependent transcriptional activation of target genes such as CAR (Reddy et al., 2024; Themeli and Sadelain, 2016). In addition to reducing toxicity to normal tissues, synNotch T cells can produce selective cytokines to regulate immune responses precisely, promote T-cell differentiation into antitumor Th1 cells, induce T cells to produce antibodies against specific immune checkpoints to enhance ICI efficacy, and trigger cancer-cell self-destruction after binding specific receptors (Li et al., 2023; Meng et al., 2025; Roybal et al., 2016). Studies also show that CAR-NKT cells can eliminate CD1d-expressing M2-type TAMs and promote epitope spreading, thereby enhancing endogenous T-cell responses to tumor-associated neoantigens (Hadiloo et al., 2023).

Another barrier is the ECM of solid tumors, which forms a physical obstacle that limits immune-cell infiltration, including CAR-T-cell infiltration. Zheng and colleagues used the synNotch receptor system to enable CAR-T cells to secrete ECM-degrading enzymes specifically at tumor sites, significantly enhancing CAR-T-cell infiltration and antitumor activity (Zheng et al., 2024). Hao et al. also developed a CAR-EV-based therapy. Because CAR-EVs are nanoscale, they can effectively penetrate solid tumors. In an HCC mouse model, CAR-TM4SF1-EVs significantly inhibited tumor growth and metastasis and prolonged mouse survival (Hao et al., 2026). In the future, combining gene-circuit design, novel effector cells, and nanocarrier technologies may improve the precision and penetration of CAR-T therapy for solid tumors and support more effective and safer personalized immunotherapy for digestive system tumors.

### 4.3 Combination with angiogenesis inhibitors

Combining Notch inhibitors with anti-VEGF agents can exert synergistic antiangiogenic effects on tumor vasculature (You et al., 2023). Anti-VEGF therapy reduces vascular density, whereas Notch inhibition, particularly targeting the DLL4/Notch axis, compromises the structural integrity and functional maturation of residual vessels (Comunanza and Bussolino, 2017; Huang et al., 2016; Lopes-Coelho et al., 2021). This dual mechanism severely disrupts tumor perfusion and induces profound, sustained hypoxia, thereby suppressing tumor growth (Miles et al., 2014). Importantly, this combinatorial strategy may overcome intrinsic or acquired resistance to VEGF inhibition by blocking the compensatory DLL4/Notch pathway, a key adaptive escape mechanism that is frequently upregulated after anti-VEGF treatment (Li et al., 2011).

Multiple human cancer xenograft models, including breast, colorectal, gastric, ovarian, and pancreatic cancer models, have shown that bispecific antibodies targeting DLL4 and VEGF have superior antitumor efficacy (You et al., 2023). Therapeutic candidates designed on the basis of this strategy include navicixizumab (OMP-305B83) (Jimeno et al., 2019), ABT-165 (Li et al., 2018), ABL001 (Yeom et al., 2020), HB-32, and HD105 (Lee et al., 2016; Zhou et al., 2019). These agents are undergoing clinical evaluation to assess safety and efficacy in patients with solid tumors. ABL001 is among the most advanced compounds in clinical development for biliary tract cancer and may later be extended to other DLL4-expressing solid tumors, including CRC, gastroesophageal junction cancer, and HCC. In the SCH GC xenograft model, HD105 also showed superior antitumor efficacy compared with monotherapy (Lee et al., 2016). In the future, bispecific antibodies may help address resistance in digestive system cancer treatment through combination with immunotherapy and through development of new immune-vascular bispecific agents. Combined targeting of DLL4/Notch and Ephrin-B2/EphB4 signaling also shows a significant cumulative effect in inhibiting tumor growth and improves treatment safety, providing another potential candidate strategy for cancer treatment (Djokovic et al., 2010).

### 4.4 Combination with other targeted pathway inhibitors

During initiation and progression of digestive system tumors, Notch signaling does not operate in isolation. Instead, it engages in extensive crosstalk with multiple signaling pathways (Shi et al., 2024; Xiang et al., 2026). This crosstalk occurs mainly in two ways: non-canonical Notch signaling can modulate downstream effectors to activate alternative pathways, and Notch signaling can be co-activated with other oncogenic pathways within the same TME. These interpathway interactions provide a mechanistic rationale for combining Notch inhibitors with agents that target complementary signaling axes.

Notch signaling maintains cell proliferation, growth, and metabolism by driving PI3K pathway signal transduction, with mTOR acting as a downstream molecule of the PI3K pathway (Piha-Paul et al., 2015). Combined Notch and mTOR inhibition may enhance therapeutic effects by directly targeting tumors and blocking tumor-dependent angiogenesis. For example, Notch signaling promotes cholangiocarcinoma development through the mTORC1 pathway, suggesting that simultaneous inhibition of Notch and mTORC1 may have synergistic antitumor effects (Namikawa et al., 2023). Peng et al. showed that combined treatment with the Notch1 inhibitor DAPT and the PI3K/Akt inhibitor LY294002 significantly suppressed EMT marker and MMP-9 expression. This indicates cooperative inhibition of EMT and ECM degradation and further reduced GC lung metastasis in a nude mouse model (Peng et al., 2020). In a related context, targeting aberrant upstream activators of PI3K/Akt signaling has also shown promise. Chen et al. recently reported that secreted CEA binds KRT1 to activate PI3K/Akt signaling and confer oxaliplatin resistance in GC, and that the small-molecule inhibitor evacetrapib restores chemosensitivity by competitively disrupting this interaction *in vitro* and in organoid models (Chen Y. et al., 2025). This finding suggests that combining evacetrapib with Notch inhibitors or chemotherapeutic agents may be a rational strategy to overcome resistance, although further studies in digestive system cancers are needed.

Activation of WNT signaling can upregulate Notch signaling, indicating that combined inhibition of WNT and Notch pathways may produce synergistic therapeutic effects (Fendler et al., 2020). Simultaneous targeting of Notch and Wnt/beta-catenin signaling has been proposed as a feasible potential treatment strategy for triple-negative breast cancer (TNBC) (Nasser et al., 2021). These two pathways are also closely related to sorafenib resistance in HCC (Yang et al., 2021), and combined use of the Notch inhibitor RO4929097 (RO) and the Wnt inhibitor XAV939 (XAV) can partially reverse resistance of colon cancer cells to 5-FU and OxaPt (Kukcinaviciute et al., 2018). Recent research further showed that adavivint, a drug that improves chemotherapy resistance in CRC, specifically reduces Notch2 protein levels and ultimately inhibits transcription of Wnt target genes, including MYC and JUN. This suggests that targeting this Notch pathway can effectively intervene in Wnt signaling and enhance therapeutic effects (Xiang et al., 2025b). Regarding interaction between Notch and JAK2/STAT3 signaling, combined treatment with the Notch inhibitor GSI IX and the JAK2 inhibitor AG-490 significantly inhibited proliferation, migration, and invasion of PDAC cells, supporting this combination as a potential therapeutic strategy for PDAC (Palagani et al., 2014).

Many studies suggest that more effective treatment strategies for digestive system tumors may be achieved by combining inhibitors that target different signaling pathways or different stages of the same pathway. However, not all pathway-inhibitor combinations produce synergy. For example, one preclinical study evaluated co-administration of MAPK and Notch inhibitors with chemotherapy in animal models but found no significant synergistic enhancement (Spartalis et al., 2019). Rational combination therapy therefore requires mechanistic understanding of context-specific crosstalk and functional interdependencies among the targeted pathways.

## 5 Conclusions and future perspectives

In digestive system cancers, Notch signaling shows context-dependent duality and can function as either an oncogene or a tumor suppressor. Its most consequential impact may lie in remodeling the TME. Dysregulated Notch signaling in tumor cells and stromal components can reprogram immune-cell function, modulate tumor angiogenesis, and drive ECM remodeling, collectively fostering a tumor-permissive microenvironment that supports growth and metastasis. Therapeutically, Notch modulators hold promise for disrupting tumor-stroma crosstalk. Concurrent use of Notch modulators with targeted anti-TME drugs may also provide synergistic therapeutic benefit. Nevertheless, the underlying molecular mechanisms and quantitative efficacy of these approaches require rigorous preclinical and clinical evaluation.

A major future challenge is the pronounced tissue and cellular heterogeneity of Notch signaling. Notch expression and function vary across digestive system cancer types, between patients, and within different regions of the same tumor. This heterogeneity directly shapes the design of targeted therapeutic strategies. The same Notch modulator may exert opposing effects across digestive cancer types. For example, GSIs combined with gemcitabine showed no clinical benefit in PDAC trials, whereas GSIs synergized with 5-FU to induce cytotoxic effects in GC cells (Martínez-Gascueña et al., 2026). Even within a single tumor type, the role of Notch signaling is not fixed. GSIs suppressed growth of the epithelial cell adhesion molecule-positive fraction in hepatoma cells (Kawaguchi et al., 2016). However, in hepatocyte subpopulations with inactivation of the RB tumor suppressor pathway, global Notch activation can inhibit HCC occurrence and development (Song et al., 2021). A one-size-fits-all approach is therefore unlikely to succeed. Therapeutic strategies should instead be context dependent. Notch inhibitors should be reserved for malignancies in which Notch clearly drives tumorigenesis, whereas Notch agonists may be needed in tumors where Notch signaling suppresses progression. Cellular heterogeneity also calls for targeted delivery systems, such as nanoparticles modified with antibodies that recognize tumor-specific surface markers. This approach could help avoid off-target modulation of immune or stromal cells that might unintentionally promote a pro-tumorigenic microenvironment.

Future research should leverage scRNA-seq and spatial transcriptomics to deconvolute this complexity. ScRNA-seq provides important insight into TME heterogeneity, whereas spatial transcriptomics integrates gene-expression data with spatial information and is crucial for understanding interactions among TME components (Heumos et al., 2023; Verona et al., 2025). Developing validated prognostic and predictive biomarkers will also be essential for patient stratification. Such biomarkers could identify patients whose tumors display either a Notch-dependent or Notch-suppressive phenotype and thereby guide personalized decisions to inhibit or activate Notch signaling. In addition, the gut microbiota has been reported to regulate immune cells and affect tumor progression through classical pathways, including Notch signaling (Troll et al., 2018). Exploring the interplay between the gut microbiota and Notch signaling in intestinal cancers therefore represents a promising frontier for modulating the local TME and improving therapeutic outcomes. Together, these strategies could guide next-generation, context-specific Notch therapeutics and support more effective personalized treatment paradigms in digestive oncology.

## Glossary

CRC: Colorectal cancer
TME: Tumor microenvironment
MDSCs: Myeloid-derived suppressor cells
TAMs: Tumor-associated macrophages
TANs: Tumor-associated neutrophils
Tregs: Regulatory T cells
DCs: Dendritic cells
DSL: Delta/Serrate/Lag-2
NICD: Notch intracellular domain
RBPJ: Recombination signal binding protein for immunoglobulin kappa J region
HCC: Hepatocellular carcinoma
EMT: Epithelial-mesenchymal transition
UBE2C: Ubiquitin-conjugating enzyme E2 C
GC: Gastric cancer
JAG: Jagged
PDAC: Pancreatic ductal adenocarcinomas
DLL4: Delta-like 4
DLK1: Delta-like 1 homolog
GSIs: γ-secretase inhibitors
MDSCs: Myeloid-derived suppressor cells
BM: Bone marrow
DC: Dendritic cells
CSF: Colony-stimulating factors
ESCC: Esophageal squamous cell carcinoma
GSEA: Gene set enrichment analysis
TIMP: Tissue inhibitor of metalloproteinases
PMN: Pre-metastatic niche
TAMs: Tumor-associated macrophages
kclTAM: Kupffer cell-like TAM
TANs: Tumor-associated neutrophils
NETs: Neutrophil extracellular traps
Tregs: Regulatory T cells
tTreg: thymus-induced Treg cells
pTreg: Peripheral-induced Treg cells
PD-1: Programmed cell death protein 1
Foxp3: Forkhead box P3
ESAM: Endothelial cell-selective adhesion molecule
CAFs: Cancer-associated fibroblasts
OSCC: Oral squamous cell carcinoma
NHDFs: Normal human dermal fibroblasts
USP5: Ubiquitin specific peptidase 5
LSD1: Lysine-specific demethylase 1
GBC: Gallbladder cancer
lncRNA: Long non-coding RNA
ECM: Extracellular matrix
CAC: Colitis-associated cancer
ROS: Reactive oxygen species
ICIs: Immune checkpoint inhibitors
PD-L1: Programmed death-ligand 1
SLFN11: Schlafen Family Member 11
PC: Pancreatic cancer
CAR-T: Chimeric antigen receptor T-cell
SynNotch: Synthetic Notch
TNBC: Triple-negative breast cancer
